# Combined Antifungal Resistance and Biofilm Tolerance: the Global Threat of Candida auris

**DOI:** 10.1128/mSphere.00458-19

**Published:** 2019-07-31

**Authors:** Ryan Kean, Gordon Ramage

**Affiliations:** a Department of Biological and Biomedical Sciences, School of Health and Life Sciences, Glasgow Caledonian University, Glasgow, United Kingdom; b Oral Sciences Research Group, School of Medicine, Dentistry and Nursing, College of Medical, Veterinary and Life Sciences, University of Glasgow, Glasgow, United Kingdom; Carnegie Mellon University

**Keywords:** *Candida*, antifungal resistance, biofilms, tolerance

## Abstract

The enigmatic yeast Candida auris has emerged over the last decade and rapidly penetrated our consciousness. The global threat from this multidrug-resistant yeast has generated a call to arms from within the medical mycology community. Over the past decade, our understanding of how this yeast has spread globally, its clinical importance, and how it tolerates and resists antifungal agents has expanded. This review highlights the clinical importance of antifungal resistance in C. auris and explores our current understanding of the mechanisms associated with azole, polyene, and echinocandin resistance. We also discuss the impact of phenotypic tolerance, with particular emphasis on biofilm-mediated resistance, and present new pipelines of antifungal drugs that promise new hope in the management of C. auris infection.

## Abstract (precis)

The enigmatic yeast Candida auris has emerged over the last decade and rapidly penetrated our consciousness. The global threat from this multidrug-resistant yeast has generated a call to arms from within the medical mycology community. Over the past decade, our understanding of how this yeast has spread globally, its clinical importance, and how it tolerates and resists antifungal agents has expanded.

## INTRODUCTION

The emergence of new microbial pathogens combined with escalating rates of antimicrobial resistance (AMR) continue to pose a global threat. The field of medical mycology is acutely aware of this since the emergence of the pathogenic yeast Candida auris, a member of the Metschnikowiaceae family. In the decade since its discovery in 2009 (1), it has quickly emerged as a prolific nosocomial pathogen, causing infections across all inhabited continents. A retrospective review of strain collections suggests that it first appeared in South Korea in 1996 (2). The simultaneous and unprecedented emergence of genetically distinct clades of the species has mystified the scientific and medical mycology communities. C. auris possesses the “superbug”-like traits typically associated with common hospital-acquired infections, such as methicillin-resistant Staphylococcus aureus (MRSA), in that it can often be multidrug resistant (MDR) and can survive and persist in the nosocomial environment. To exacerbate matters, unambiguous identification of this yeast remains difficult, further emphasizing the organism as a global health threat.

At the writing of this article (June 2019), C. auris had been reported in 33 countries on 6 different continents. Crude mortality rates are varied but have been reported to be as high as 66% (3). Whole-genome sequencing has revealed four geographically and phylogenetically distinct clades of the organism (South American, African, South Asian, and East Asian), which contain almost genetically identical strains within clades but can harbor tens of thousands of single nucleotide polymorphism (SNP) differences between clades (4). Interestingly, every clade, with the exception of the East Asian clade, has been associated with outbreaks and invasive infections. In a recent study of isolates from South Korea, cases associated with the East Asian clade are almost uniquely (>93%) associated with ear infections (2). The enigma of the origin and evolution of C. auris is perplexing and not currently known. Casadevall and colleagues recently proposed an interesting and controversial hypothesis that the emergence of C. auris could potentially be the first example of a fungal pathogen to emerge as a consequence of climate change (5). The authors postulate that based on their phylogenetic and thermotolerance analysis of C. auris, the increase in ambient temperatures as a result of global warming may have acclimatized the organism to adapt to and survive at avian and mammalian temperatures, with transmission from birds to rural areas being a potential mechanism of its emergence. However, this is highly speculative, and it is likely that many other factors are involved, which warrants further study.

The principal factor that makes this organism so enigmatic is its intrinsic resistance to conventional front-line antifungal agents and its tolerance to antiseptics and disinfectants. Its resilience and adaptivity within a variety of clinical environments have afforded it with the opportunity to emerge and cause alarm among medical professions worldwide. This review will focus on exploring the factors driving its resistance to therapeutic management that we have uncovered over the first decade of its discovery.

## ANTIFUNGAL RESISTANCE: FROM CLINIC TO LABORATORY

**FIG 1:**
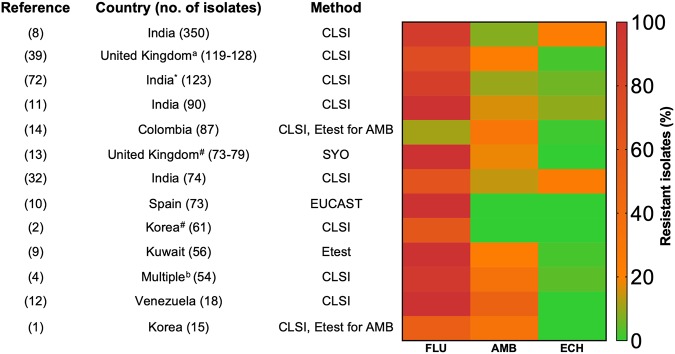
Geographic antifungal resistance rates of Candida auris. Heatmap depicts the percentage of resistant isolates to fluconazole (FLU), amphotericin B (AMB), and echinocandins (ECH), and numeric values of resistance are given in Table S1. *, the echinocandin is anidulafungin and not caspofungin; #, the echinocandin is micafungin; ^a^, includes isolates from other countries; ^b^, isolates from Pakistan, India, South Africa, and Venezuela.

According to a recent review by Lockhart, when it comes to C. auris, resistance is the new norm (6), with a significant minority of clinical isolates displaying antifungal susceptibility. Resistance has been reported across all main classes of antifungals, with a worryingly high proportion of isolates being documented as multidrug resistant. No definitive MIC breakpoints exist, but tentative breakpoints have been suggested by the Centers for Disease Control and Prevention (https://www.cdc.gov/fungal/candida-auris/c-auris-antifungal.html) and are supported by studies in a neutropenic mouse model to assess antifungal target ranges (7). Using these guidelines, the rates of resistance to fluconazole (FLU), amphotericin B (AMB), and echinocandins (ECH) (caspofungin, anidulafungin, and micafungin) are presented in Fig. 1. Most notably, C. auris is frequently associated with high levels of FLU resistance, with multiple studies reporting resistance in over 90% of isolates (4, 8–13). The heatmap clearly illustrates this point, where high rates of resistance (red/orange) are pronounced across the globe, though notably low rates of FLU resistance (11%) have also been reported in Colombia and South Korea (14). AMB resistance, although not as pronounced as FLU resistance, is also a significant issue because AMB resistance is extremely rare in other fungi, as it is thought to come at a fitness cost to the organism (15). Resistance rates typically range up to 30% (4, 14), with a small study from Venezuela having 50% resistant isolates (12). Several studies have demonstrated elevated MICs for this polyene, ranging from 2 to 4 μg/ml, though these are not as high as observed for other members of the Metschnikowiaceae family (16 μg/ml), which includes Candida haemulonii, Candida duobushaemulonii, and Candida pseudohaemulonii (16). Interestingly, resistance to AMB within a Colombian isolate cohort was shown to be geographically related, with resistance significantly associated with hospital outbreaks in the northern region of the country compared to the central region (14). It should be noted that the platform used to test AMB can have a bearing on sensitivity, as it has been reported that the Vitek AST-YS07 card can provide misleading and elevated AMB MICs (11). Therefore, standard CLSI broth microdilution testing may be more a more accurate and reliable measure. Echinocandin resistance remains limited, but unfortunately, it has been reported alongside other antifungal resistance phenotypes. In one of the largest multicenter (10 hospitals) studies to date, the resistance rates of 350 isolates collected from between 2009 and 2017 in India were reported (8). Here, 2% of the isolates were resistant to echinocandins (>8 μg/ml), alongside 8% resistant to AMB (>2 μg/ml) and 90% resistant to FLU (32 to ≥64 μg/ml). Although termed as fungicidal agents against the majority of *Candida* spp., a recent study of Columbian isolates revealed that both anidulafungin and caspofungin are in fact fungistatic against C. auris *in vitro* (17). Assessment of echinocandin sensitivity should be exercised with caution due to the paradoxical effect observed with caspofungin; instead, micafungin or anidulafungin should be used for testing (18). The factors driving echinocandin resistance are hospitals where these antifungals are recommended as a first-line treatment or where other antifungals have failed. Irrespective, the emerging landscape for C. auris antifungal resistance is the development of multiresistance phenotypes, driven by prior antifungal exposure and sequential antifungal treatment failures (19–21). However, despite this pessimistic viewpoint, there appears to be clade-specific sensitivity/resistance profiles; in India, for example, there are significant levels of fluconazole sensitivity, whereas, other geographical sites have high levels of multiazole resistance (6). In order to mitigate the development of multidrug resistance, several *in vitro* studies have investigated the possibility of antifungal combinations. Indeed, the combination of micafungin with voriconazole was shown to exhibit synergistic effects (22), as has also been reported for sulfamethoxazole-azole combinations (23). These prove the possibility that C. auris infections can be managed effectively, though in order to fully develop effective antifungal strategies, we must understand what enables C. auris to withstand and respond to our antifungal arsenal.

10.1128/mSphere.00458-19.1 TABLE S1 Published antifungal susceptibility data for Candida auris. *, the echinocandin is anidulafungin and not caspofungin; #, the echinocandin is micafungin; ^a^, isolates from other countries; ^b^, isolates from Pakistan, India, South Africa, and Venezuela. Download Table S1, DOCX file, 0.03 MB. Copyright © 2019 Kean and Ramage. 2019 Kean and Ramage This content is distributed under the terms of the Creative Commons Attribution 4.0 International license.

## OUR CURRENT UNDERSTANDING OF ANTIFUNGAL DRUG MECHANISMS

**FIG 2:**
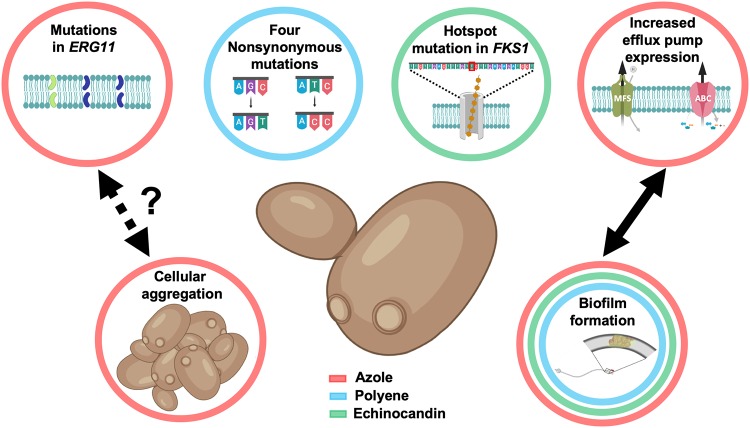
Conventional and phenotype-derived resistance mechanisms of Candida auris. Shown are various genetic and phenotypic resistance mechanisms employed by C. auris. Circles indicate resistance to azoles (red), polyenes (blue), and echinocandins (green). Figure was created with BioRender.

Antifungal resistance is generally driven by several factors, including point mutations of the cellular target, overexpression of target molecules, and efflux pump extrusion of antifungals (24). Mechanistically, the factors underpinning C. auris resistance have become more apparent as we invested more resources to develop our understanding, learning what we can from Candida albicans. Figure 2 illustrates some of our current understanding of C. auris resistance mechanisms. Indeed, the first multi-omics study comparing a clinically resistant (to FLU and ECH) isolate and a sensitive C. auris isolate, alongside C. albicans, revealed drug resistance profiles distinct from one another, with major differences in efflux pumps, cell wall, sterols, carbon utilization, glycerolipids, and sphingolipids collectively playing a role in resistance, albeit in a limited study of only two isolates (25). Moreover, the resistant isolate displayed an enhanced biofilm proteome profile, a phenotype associated with adaptive resistance. Comparing these isolates to Candida albicans ATCC 90028, it was shown that both C. auris isolates had major differences with this regarding their carbon utilization and downstream lipid and protein contents. Taken together, these data indicate that C. auris displays a species-specific resistance profile.

Azole resistance is commonly mediated by amino acid substitutions in the lanosterol 14-alpha-demethylase (*ERG11*) gene, comprise multiple variant substitutions, including Y132F, K143R, and F126L, and appear to be clade related (4). Heterologous expression of C. auris *ERG11*-Y132 and *ERG11*-K143R alleles in Saccharomyces cerevisiae exhibited increased MICs to both FLU and voriconazole. In contrast, heterologous expression of two substitutions (I466M and Y501H) identified in isolates from the South American clade showed no effect on azole susceptibility (26). In addition, it has also been shown that exposure to FLU can increase the expression of *ERG11* up to 7-fold (8), and that an increased copy number of *ERG11* may also contribute to resistance (27). Indeed, a subsequent investigation demonstrated that transient gene duplication of *ERG11* and *CDR1* has the capacity to increase tolerance to FLU in older cells (28), an *in vitro-*inducible upregulation of *ERG11* validated elsewhere (8). Expanding our mechanistic insights, recent work by Cowen’s group has implicated a role of the molecular chaperone Hsp90 for tolerance to azoles, whereas the efflux pump gene *CDR1* played a distinct role in high-level azole resistance that was revealed from deletion studies that abrogated resistance (29). Corroborating these studies, efflux pump-mediated resistance through an ATP-binding cassette (ABC) and the major facilitator superfamily (MFS) has been shown to be significantly greater in a panel of C. auris isolates than in Candida glabrata and *C. haemulonii* isolates (16). A recent study from Rybak et al. aimed to functionally characterize the role for efflux pumps in triazole resistance in C. auris (30). They demonstrated that the transcript levels of the *CDR1* and *MDR1* genes were increased in triazole-resistant isolates in comparison to a fluconazole-susceptible isolate. Using a Cas9-ribonucleoprotein (Cas9-RNP) transformation system, they were able to show that a Δ*cdr1* mutation in a resistant isolate was able to increase susceptibility to fluconazole and itraconazole by 64- and 128-fold, respectively, with notable reductions in MIC also demonstrated in other azoles. The function of efflux pumps in azole resistance appears to be predominantly associated with *CDR1*, as analysis of the Δ*mdr1* mutant showed minimal effect on increasing azole sensitivity (30), which is in line with the aforementioned molecular analyses (29). Functional assays performed in biofilms also support these findings (31).

Unlike azoles, the mechanism(s) governing resistance to polyenes are poorly understood in *Candida* species in general. Depletion of ergosterol composition through loss-of-function mutations is thought to be the primary resistance mechanism in a limited number of species, with *erg3* mutations thought to be important in cross-resistance (24). Whole-genome sequencing of resistant isolates identified four novel nonsynonymous mutations, highlighting a potential association with resistance. These mutations included those in genes with homology to the transcription factor Flo8 gene of C. albicans and a membrane transporter (14). To understand the mechanisms responsible for amphotericin B resistance, Muñoz and colleagues performed comparative transcriptional analysis on a resistant isolate and a sensitive isolate after exposure to these drugs (27). Using RNA sequencing (RNA-seq), it was shown that 106 genes were induced in response to AMB in the resistant isolate (27). Notably, genes involved in the sterol biosynthetic process were identified, of which genes involved in the ergosterol biosynthesis pathway were highly induced (*ERG1*, *ERG2*, *ERG6*, and *ERG13*), which logically correlates with the maintenance of cell membrane stability. A limitation of this entire approach is the comparative genomic nature, which may limit our future discovery and understanding of novel antifungal-resistant genes specific to amphotericin B and other agents.

Echinocandins are considered the first-line therapy for invasive infections, though multiple studies have reported the isolation of resistant isolates across various geographical regions, with the highest levels of resistance reported in India (8, 32). Resistance is typically associated with hot spot mutations in the *FKS1* gene, which encodes the 1-3-β-glucan synthase enzyme, the target of echinocandins, resulting in lower affinity of the enzyme to the drug (8). A study by Kordalewska and coworkers identified that an S639F mutation in *FKS1* conferred pan-echinocandin resistance in four Indian isolates (18), with another study identifying a different amino acid substitution in the same position (S639P) (33). Here, the interpretation of the paradoxical effect becomes pertinent, as the high *in vitro* concentrations were not shown to correlate *in vivo* with isolates not harboring the *FKS1* mutation (18).

## PHENOTYPE-DERIVED ANTIFUNGAL TOLERANCE

To combat both host and environmental stressors and selectively adapt to the surrounding microenvironment, pathogenic fungi often employ unique phenotypes that confer an advantage to colonize the surrounding environment. Phenotypic plasticity has been extensively described in C. albicans, with the ability of the organism to morphologically transform between yeast and hyphae and also switch between white and opaque cell types the best understood mechanisms (34, 35). Phenotypic behavior in C. auris is less well understood; however, various phenotypes have recently been described that have been shown to have implications for antifungal resistance. A recent study by Bhattacharya and colleagues demonstrated that transient gene duplication as a result of replicative aging confers increased antifungal resistance in C. auris (28), as has previously been described in C. glabrata and Cryptococcus neoformans (36, 37). The authors showed that older C. auris cells of 10 generations displayed increased tolerance across the four main classes of antifungals compared to that of a younger cell equivalent (0 to 3 generations). Using FLU-susceptible isolates, it was shown that with older generations of these isolates, cells could survive FLU concentrations of 256 μg/ml, as well as be unresponsive to treatment *in vivo* using Galleria mellonella. This decreased susceptibility was shown to be responsible for both increased expression and gene duplication of *CDR1* and *ERG11*. Another phenotypic difference which has been shown in C. auris is through cellular aggregation. This phenomenon was first identified in clinical isolates from the United Kingdom (38). It was shown that a subset of isolates displayed an aggregative phenotype that could not be physically or chemically disrupted with detergent. Interestingly, these aggregates were shown to be significantly less virulent in a Galleria mellonella model, with single-celled isolates exhibiting virulence comparable to that of C. albicans. A recent study from the same authors has now identified that the ability to aggregate is indeed an inducible trait that can be stimulated with prior exposure to triazole and echinocandin antifungals (39). These findings were based upon the initial observation that isolates from the South African clade naturally form aggregates and exhibit escalated MICs to triazoles. Exposure of isolates to even low concentrations of triazoles and anidulafungin induced aggregate formation in single-celled isolates of the South Asian clade but could not be induced by polyenes or flucytosine. The mechanism by which cells aggregate to survive is unknown, but the authors speculate that it could be linked to a stress response to perturb ergosterol synthesis. The clinical implications of aggregation with regards to antifungal resistance and environmental survival remain limited, but large aggregates of cells have been recovered from harvested tissue of a murine model, suggesting that it may be used as a strategy to withstand host defenses (16). A recent study comparing phenotypes of isolates from either colonizations or systemic infections identified the ability to aggregate with a statistically significant association with colonization, suggesting a role of this phenotype in persistence (40). More recently, we reported that aggregating phenotypes enhance environmental survival through enhanced adhesion and biofilm formation and recalcitrance to sodium hypochlorite treatment (41).

Biofilm formation is a well-studied mechanism by which many microbial pathogens can confer increased resistance and tolerance to antimicrobials. Numerous pathogenic fungi possess the ability to exist as these communities, with C. albicans and Aspergillus fumigatus being the most extensively studied (42, 43). The ability of C. auris to form biofilms was initially dismissed, although the semiquantitative methods used to draw these conclusions were rudimentary (44). Clinically, C. auris has been recovered from a variety of indwelling medical devices, including catheters, line tips, and neurological shunts (16, 38, 45), as well as multiple fomites in the nosocomial environment and reusable temperature probes (13), suggesting a role for biofilm lifestyle in both the host and the environment. Indeed, more recent, in-depth analyses from a number of different groups have demonstrated the potential of this pathogen to form antifungal-tolerant biofilms (31, 46–50). A study by Sherry and colleagues first identified that C. auris was able to produce intermediate quantities of biomass compared to C. albicans and C. glabrata. Despite not producing biofilms as robust as those of C. albicans, these communities were shown to tolerate the three major classes of antifungals, including amphotericin B and micafungin, the recommended therapy for C. albicans biofilm infections. Expanding on these findings, our group performed transcriptional analysis to determine the underlying mechanisms associated with biofilm development and tolerance (31). We demonstrated that tolerance to all classes of antifungals was biofilm-phase dependent, with mature biofilms (24 h) exhibiting resistance to all three classes of antifungals. Correlating with this was the increased expression of a number of genes encoding efflux pumps of both ABC and major facilitator superfamily (MFS) transporters. Inhibition of these transporters was shown to improve the susceptibility of biofilms to fluconazole by 4- to 16-fold. Also upregulated during biofilm formation were multiple glucan-modifying genes, which have key roles in biofilm extracellular matrix formation. The matrix of C. auris has been shown to be both biochemically and functionally similar to those of other *Candida* spp. in that is primarily composed of glucan and mannan polysaccharides that can sequester azole antifungals (46). These two mechanisms are conserved across biofilms formed by other *Candida* species and likely explain the increased tolerance to azoles. Whether these observed mechanisms also contribute to polyene and echinocandin tolerance associated with C. auris biofilms individually or in combination remains unknown. However, given that these antifungals are active against C. albicans biofilms, it is plausible to speculate that other novel mechanisms may be involved, and as such, further work is required.

## IMPLICATIONS FOR INFECTION CONTROL

Given the well-documented resistance profile of this pathogenic yeast and its capacity to cause hospital outbreaks and high associated mortality rates, infection control within the nosocomial environment is crucial. Multiple reports have suggested that despite the implementation of more stringent infection control measures, that cases of C. auris colonization and associated infections can continue (13). Unlike the majority of other major *Candida* species which typically inhabit the gut and oral cavity, C. auris readily colonizes the skin, which can be problematic for acquired nosocomial infections. In addition, viable cells have also been recovered from various surfaces within the environment, including bedding and flooring, as well as reusable equipment, such as axillary temperature probes (13, 51). *In vitro* survival studies have shown that the organism can survive for up to 28 days on different abiotic substrates, including plastic and steel (52, 53).

Various studies have assessed the efficacy of routinely used commercially available biocides against C. auris and have demonstrated optimal efficacy against the organism, with few exceptions (54–57). Quaternary ammonium-based agents have been shown to have minimal efficacy against C. auris (55) and were used to clean reusable equipment associated with an outbreak in Oxford (13). In addition, chlorhexidine has also shown varied formulation-dependent efficacy, with resuspension of the active in 70% isopropyl alcohol yielding more significant killing at a short contact time (56). It is worth noting that the majority of disinfection studies have focused on testing in suspension, which does not take into consideration the complex topographies of substrates and surface-induced phenotypes employed by this organism. Adherent C. auris cells to various hospital fomites have been shown to demonstrate reduced efficacy against clinically relevant concentrations of sodium hypochlorite and peracetic acid (58). The mechanisms employed to survive and persist in the environment are relatively unknown, but there is mounting evidence to suggest that biofilm formation may be a contributing factor, as has been suggested for MRSA (59). Ledwoch and Maillard recently demonstrated using a dry biofilm model that C. auris could withstand and be recovered following treatment with various biocides, in addition to showing significant transferability to sterile surfaces (49). These findings are in agreement with those of our recent study highlighting the expression of biofilm-associated characteristics after 14 days of survival (41). It was also shown that the aggregative phenotype may have a function in environmental survival. Following treatment with sodium hypochlorite, significant quantities of aggregative cells were recovered 2 weeks posttreatment, compared to complete eradication of a single-celled isolate following treatment (41). In addition, mature C. auris biofilms grown using a three-dimensional biofilm model can tolerate both chlorhexidine and hydrogen peroxide treatment but remained susceptible to povidone iodine (60). Interestingly, the cell wall integrity pathway gene *HOG1* has been shown to regulate stress resistance, with C. auris displaying increased tolerance to hydrogen peroxide compared to C. albicans (61). These molecular approaches are therefore critical in the rationale design of new antifungals.

## CANDIDA AURIS AND THE ANTIFUNGAL PIPELINE

With the alarming emergence of antifungal resistance, there is an increasing and urgent need for the development of new antifungal therapies (62). The efficacy of the existing arsenal has plateaued in recent years and is plagued with off-target effects and bioavailability issues, coupled with the ever-increasing rise in antifungal-resistant isolates (63). There are, however, a number of improvements to existing antifungals and compounds with novel mechanisms of action which remain within the antifungal pipeline and have shown promising activity against C. auris. These molecules include expansions of current drug class targets, such as β-glucan synthase inhibitors, as well as novel mechanisms of action targeting glycosylphosphatidylinositol (GPI) protein and chitin synthesis. Ibrexafungerp (formerly known as SCY-078) is perhaps the most promising and is currently in a phase 3 clinical trial for invasive candidiasis caused by C. auris. It has been shown to have potent activity against C. auris *in vitro*, including against echinocandin-resistant isolates (64). Furthermore, it possesses documented antibiofilm activity, reducing both metabolic activity and biofilm biomass (48). The novel, long-lasting echinocandin rezafungin (CD101) has also been shown to be active against C. auris both *in vitro* and *in vivo* using a neutropenic mouse model (33, 65). It does appear, however, to have reduced susceptibility to resistant isolates containing the S639P *FKS1* hot spot mutation (33). Another potential novel candidate is MYC-053, which represents a new class of antifungals. It functions through a dual mechanism of action in which it simultaneously inhibits both intracellular nucleic acid and targets chitin synthesis of the cell wall (66). Data on its efficacy against C. auris are limited to a single study; however, it has shown efficacy against azole- and echinocandin-resistant isolates (66). Another antifungal molecule with a novel mechanism of action is APX001, which blocks GPI protein synthesis via inhibition of the cell wall transfer protein GWT1. It has been shown to have antifungal activity against resistant C. auris isolates (67), in addition to having greater *in vivo* efficacy in an invasive candidiasis murine model than anidulafungin (65).

An attractive approach to combating antimicrobial resistance is the repurposing of off-patent drug libraries. This “teaching an old dog new tricks” approach has been employed across a number of pathogens of bacterial, viral, parasitic, and fungal origins (68). Several libraries of compounds are readily available, which allows for the high-throughput, nonbiased screening of compounds to identify potential novel activities, with a number of studies already identifying molecules with antifungal activity against C. auris. Screening of 1,280 small molecules from the Prestwick Chemical library by Wall et al. identified the organoselenium compound ebselen to possess significant antifungal activity against C. auris (69). Ebselen was shown to be active against multidrug-resistant C. auris strains in both planktonic and biofilm forms, as well as a large number of other clinically relevant yeasts and molds. Another compound of interest identified from a repurposing library is alexidine dihydrochloride (70). Like ebselen, this molecule has broad-spectrum and antibiofilm activity against a number of pathogens, including C. auris. Interestingly, this study identified only 6 compounds from the repurposing library with activity against C. auris, compared to 27 that were active against C. albicans, further highlighting the resistant nature of this emerging pathogen (70). Another library screening study by de Oliveira et al. identified a number of off-patent molecules with activity against C. auris, including the aforementioned ebselen (71). Both this molecule and the antiplatelet drug suloctidil exerted synergistic activity against a panel of C. auris isolates when used in combination with voriconazole (71). Taken together, these molecules appear promising for the future control of C. auris.

## CONCLUDING REMARKS

The study of C. auris is in its relative infancy, though it has caused a sensation within the medical mycology community and beyond due to its multidrug resistance “superyeast” persona. To our advantage, we have an army of skilled and willing researchers with sophisticated multi-omic techniques that will enable us to tease out the most salient and subtle of features, ultimately identifying the Achilles heel of this tolerant yeast. It will also allow us to add to our understanding of how *Candida* spp. dynamically adapt to a wide range of environments and stressors. Existing antifungal therapies are limited and must be used cautiously when tackling culture-positive infections. Therefore, augmenting antifungal therapies with novel agents in the antifungal pipeline is our most promising of strategies, which will come to fruition over the next decade.
