# Supplementary material for: Combined Antifungal Resistance and Biofilm Tolerance: the Global Threat of Candida auris
Source: mSphere. 2019 Jul 31;4(4):e00458-19. doi: 10.1128/mSphere.00458-19 (PMC6669339; doi:10.1128/mSphere.00458-19)
Supplement: TABLE S1 [file mSphere.00458-19-st001.docx]

Supplementary Table 1. Published antifungal susceptibility data of *Candida auris*

| **Reference** | **Country (no. of isolates)** | **Resistant isolates (%)** | | |
| --- | --- | --- | --- | --- |
|  |  | **FLU** | **AMB** | **ECH** |
| ([8](#_ENREF_8)) | India (350) | 90 | 8 | 23 |
| ([39](#_ENREF_39)) | United Kingdom (119-128)^a^ | 73 | 23 | 2 |
| ([71](#_ENREF_71)) | India (123)^*^ | 86 | 10 | 6 |
| ([11](#_ENREF_11)) | India (90) | 100 | 16 | 9 |
| ([14](#_ENREF_14)) | Colombia (87) | 11 | 31 | 1 |
| ([13](#_ENREF_13)) | United Kingdom (73-79)^#^ | 100 | 18 | 0 |
| ([32](#_ENREF_32)) | India (74) | 66 | 14 | 24 |
| ([10](#_ENREF_10)) | Spain (73) | 100 | 0 | 0 |
| ([2](#_ENREF_2)) | Korea (61)^#^ | 62 | 0 | 0 |
| ([9](#_ENREF_9)) | Kuwait (56) | 100 | 23 | 2 |
| ([4](#_ENREF_4)) | Multiple (54)^b^ | 93 | 35 | 4 |
| ([12](#_ENREF_12)) | Venezuela (18) | 100 | 50 | 0 |
| ([1](#_ENREF_1)) | Korea (15) | 53 | 33 | 0 |
